# 

**Published:** 1887-02-12

**Authors:** 


### THE HOSPITAL

An Institution, Family, and Parochial Journal of
Hospitals, Asylums, & all Agencies for the Care of the Sick, Criticism & News.

### CONTENTS.

**Morphio-Mania** - 325

**Words of Consolation.**—VI. Nurses' Trials.—Weariness and Prayer - 326

**Poetry.**—In the Children's Ward - 326

**The Story of the Hospitals.**—The Cancer Hospital, Brompton - 327

**Among the Out-Patients.** By One of the Crowd - 329

**A Relic of John Hunter** - 330

**The Editor's Letter-Box.**—

The Hull Infirmary - 331

The Training of Midwives - 331

The Best Tank for Disinfecting Linen - 331

The Richmond Hospital: A Correction - 331

The Medicinal Properties of Woodall Spa Waters - 331

**Notes and News.**—Munificent Donation to Morley House.—Stock Exchange Collection.—Vacancies, etc. - 332

**Annotations.**—Sir James Picton on Homœopathy.—The Proposal to change Hospital Sunday.—Is Medical Practice Humbug? - 335

**Till the Doctor Comes.**—X. Poultices - 336

**Reminiscences of the Insane.** By a Mental Physician - 336

**A Hospital Appeal** - 337

**Workhouse Nursing: How not to do it.**—Nurses' Wages fixed by a Dutch Auction - 338

**Difficult Cases.**—Hopeless, Offensive, and Incurable.—Worthy of Sympathy and Support - 339

**The Quilter Prizes.**—Hospital Housekeeping and Management - 339

**Amusements with Profit** - 340

**The Children's Corner.**—Puzzles, Answers, etc. - 340

**In- and Out-Patient's Hospital Diary** - 341

